# Identification of enzymes responsible for the reduction of geraniol to citronellol

**DOI:** 10.1007/s13659-011-0032-6

**Published:** 2011-12-13

**Authors:** Tian-Tian Yuan, Qian-Qian Chen, Pei-Ji Zhao, Ying Zeng, Xiao-Zhu Liu, Shan Lu

**Affiliations:** 1 State Key Laboratory of Phytochemistry and Plant Resources in West China, Kunming Institute of Botany, Chinese Academy of Sciences, Kunming, 650201 China; 2 Life Science College, Southwest Forestry University, Kunming, 650224 China; 3 State Key Laboratory of Pharmaceutical Biotechnology, Nanjing University, Nanjing, 210093 China

**Keywords:** allylic alcohol, citronellol, geraniol, old yellow enzyme, reduction

## Abstract

The reduction of geraniol to citronellol is the first step for the synthesis of natural phytol in the production of tocopherols and natural vitamin K. Baker’s yeast was used in the bioreduction described above as a whole-cell biocatalyst. However, the enzyme responsible for the reduction of geraniol to citronellol is not yet known. Four old yellow enzyme (OYE) genes were cloned from yeast and plants, and expressed in *Escherichia coli* for a high level of recombinant proteins. The recombinant protein displayed a catalytic activity of converting geraniol to citronellol as a sole product verified by GC-MS analyses. The recombinant OYE2 intact cells were found to show 3.7 and 1.9-fold higher activity than that of yeast cells and the recombinant crude extracts, respectively. Compared to the recombinant fusion enzyme, the entrokinase-cleaved enzyme displayed nearly identical activity for geraniol reduction. To our knowledge, this is the first enzyme identified to catalyze the formation of citronellol from geraniol by reducing the allylic alcohol double bond, which is normally known as inactivating group for the old yellow enzymes.

**Electronic Supplementary Material:**

Supplementary material is available for this article at 10.1007/s13659-011-0032-6 and is accessible for authorized users.

## Introduction

The reduction of geraniol to citronellol is the first step for the synthesis of natural phytol in the production of tocopherols and natural vitamin K. Baker's yeast (*Saccharomyces cerevisiae*) was used in the bioreduction described above as a whole-cell biocatalyst.[1,2] However, the enzyme responsible for the reduction of geraniol to citronellol is not yet known[3]. Geraniol is considered to be a "non-activated" alkene, in contrast to the activated alkenes bearing an electron-withdrawing group, such as ketone, aldehyde, carboxylic acid, ester, anhydride, lactone, imide, nitro. Recently, enzymes from the old yellow enzyme (OYE) family have been discovered to catalyze the asymmetric reduction of activated alkenes, creating up to two chiral centers and, thus, showing a great potential in the industrial reduction of many commercially useful substrates.[3–6] The OYEs are characterized by high stereospecificity, strict regioselectivity, and rather broad substrate specificity. Fungi, particularly various yeasts, and bacteria were prominently used in whole-cell biotransformations of activated alkenes; whereas plant cells have only a minor part in the field. However, whole-cell biotransformations frequently resulted in poor product yields due to undesired side reactions such as carbonyl group reduction, ester hydrolysis, acyloin reactions, and acetyl cleavage, in addition to substrate competition and limited enzyme amount in the original cells. To avoid these problems, isolated and/or cloned enzymes have gained growing interest in biocatalytic applications. Successful attempts in the chemo- and stereoselective reduction of activated alkenes were reported by using cloned yeast OYE1 from *Saccharomyces carlsbergensis*,[5–7] OYE2 and OYE3 from *S. cerevisiae*,[6–8] CmOYE from *Candida macedoniensis*;[9] and bacterial OYE homologues YqjM from *Bacillus subtilis*, NCR from *Zymomonas mobilis*;[6,10] and plant OYE homologues LeOPR1 and LeOPR3 from *Lycopersicon esculentum* (tomato).[10,11] The plant 12-oxophytodienoate reductase (OPR) is involved in the reduction of (9*S*,13*S*)-12-oxophytodienoate to the corresponding cyclopentanoic acid OPC-8:0 for jasmonate biosynthesis.[12] By using the cloned OPR1 and OPR3 from tomato, a striking change of stereoselectivity and a remarkably broad substrate spectrum were achieved for asymmetric reduction.[10,11] Although the above OYE enzymes have been biochemically well characterized, they have never been investigated for the capacity of reducing the allylic alcohol double bond in geraniol, because alcohols are not typically favored as activating groups for OYEs.[3] In our effort to broaden the applicability of plant OPR, we came across its catalytic activity of converting geraniol to citronellol as a sole product (Scheme).

*To whom correspondence should be addressed. E-mail: biochem@mail.kib.ac.cn

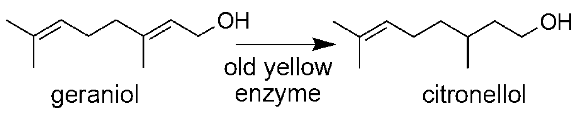

**Scheme.** The enzyme-catalyzed reduction of geraniol to citronellol.

**Results and Discussion**

Two novel OPR homologues designated as *EaOPR* and *IeOPR*, together with the known *HbOPR* from rubber tree and *OYE2* from *S. cerevisiae* were cloned and functionally expressed by pET32a+ in *E. coli* BL21 (DE3). The complete cDNAs for *EaOPR* [GenBank: JN828575] and *IeOPR* [GenBank: JN828576] were recovered from the perennial herbs *Epimedium acuminatum* (Berberidaceae) and *Isodon eriocalyx* (Lamiaceae) through degenerate primers and subsequent rapid amplification of cDNA ends (RACE). Based on SDS-PAGE analyses, higher level of soluble protein expression was obtained with 0.1 mM isopropyl-*β*-D-thiogalactopyranoside (IPTG) at 20°C for 23 h (Figure S1 in Supplementary Material). Crude extracts of the induced cells were assayed for the C=C bond reductase activity using methyl crotonate, methyl *trans*-cinnamate, 3-methyl-2-cyclohexenone, and geraniol as a substrate. Significant enzymatic production of the predicted product was observed when geraniol was incubated in the presence of each crude enzyme; and citronellol was detected as a sole product by GC-MS with authentic compound. Incubating with 3-methyl-2-cyclohexenone, only the recombinant HbOPR and OYE2 led to formation of 3-methyl-2-cyclohexanone as detected by GC-MS (Figure S2 in Supplementary Material). This activity was previously observed for the recombinant OYE2.[6] All the recombinant enzymes were inactive to methyl crotonate and methyl *trans*-cinnamate based on GC-MS analyses (data not shown).

The purified recombinant HbOPR was used for further characterization of enzyme properties. After purification under native condition on Ni-NTA His•Bind Superflow, the analysis of the elute on SDS-PAGE led to the detection of one band corresponding exactly to the predicted size of the recombinant protein of approximately 60 kDa (Figure 1A). When incubated with geraniol and cofactor NADH or NADPH, the purified HbOPR made citronellol as a primary product. The product peak at 9.31 min (retention time) generated the dominant mass segments at *m/z* 41, 55, 69, 81, 95, 123 and 156 perfectly matching the authentic citronellol in retention time and mass spectrum from GC-MS analyses (Figure 1B and C). In addition, the recombinant HbOPR is less specific to nicotinamide cofactors, appearing to accept either NADH or NADPH about equally well (data not shown). This is in contrast to carbonyl reductases which show a clear preference for either NADH or NADPH.[13]

Based on the relative activity estimated from the GC integral ratio of the product citronellol (**1**) to the internal standard (**3**), the recombinant HbOPR and OYE2 were found to be most active to geraniol reduction among the four recombinant enzymes studied here (Figure 2A and B). The lowest activity was found for the recombinant EaOPR from the herb *Epimedium acuminatum*. As shown in Figure 2B, cell lysates from the induced BL21 (DE3) with or without the

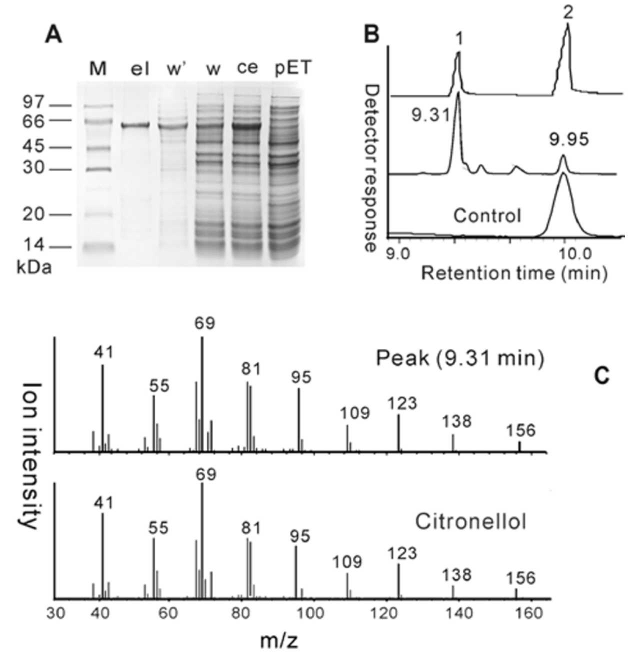

**Figure 1.** (**A**) Purification of the His-tagged proteins analyzed by SDS-PAGE. **ce**, crude enzyme extract before purification; **el**, eluate of the purified protein; **w** or **w′**, fractions collected with washing buffer of 20 mM Tris-HCl pH7.5 containing 0.5 M NaCl and 5 mM or 50 mM imidazol; **M**, protein size marker; **pET**, extracts of *E. coli* with original pET32a+ as the control. (**B** and **C**) GC-MS analyses of the authentic citronellol (**1**) and geraniol (**2**), and the reaction product formed by the recombinant HbOPR and the inactive enzyme as control, with geraniol as substrate. GC profiles (**B**) and the corresponding mass spectra (**C**) illustrate the product peak (9.31 min of retention time) matching citronellol (**1**) with respect to retention time and mass fragmentation pattern.

original pET32a+ displayed a very low activity as background (Table S2 and Figure S3 in Supplementary Material). In addition, the results of recombinant OYE2 were compared with those obtained by employing baker's yeast intact cells. For the estimation we used equal amount of *S. cerevisiae* yeast cells, the recombinant OYE2 intact cells, and the recombinant OYE2 crude extracts from the same amount of OYE2 cells. Results demonstrated that the recombinant OYE2 intact cells were best performer, showing 3.7 and 1.9-fold higher activity than that of yeast cells and crude extracts, respectively (Figure 2C, also see Table S2 and Figure S4 in Supplementary Material). Similar results were observed when the reaction mixtures were incubated at 28°C (Table S2).

To further clarify the role of the fusion tag in the recombinant proteins, enzymatic cleavage was carried out with entrokinase upon the recombinant HbOPR fusion protein. A complete cleavage was observed for the protein incubating with 40 IU of entrokinase at RT for 2 h, yielding the fusion tag and the 'net' HbOPR of approximately 44 kDa (Figure 3A). Compared to the fusion HbOPR, the 'net' HbOPR enzyme possessed nearly identical activity for geraniol reduction (columns a and b, Figure 3B), reducing geraniol to citronellol as a primary product. By subtracting the relative activity of the

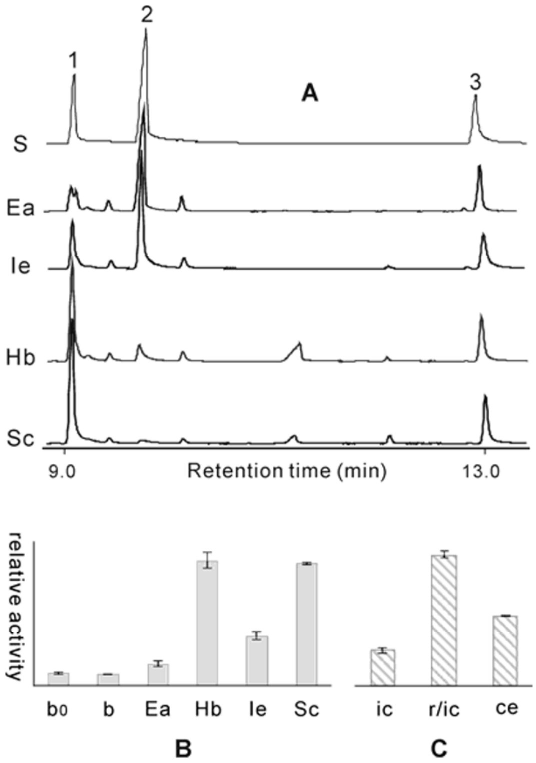

**Figure 2.** (**A**) GC profiles of the authentic citronellol (**1**), geraniol (**2**), the internal standard methyl *trans*-cinnamate (**3**) as standards (**S**), and the recombinant crude enzyme assay for EaOPR (**Ea**), IeOPR (**Ie**), HbOPR (**Hb**), OYE2 (**Sc**), respectively. (**B**) Crude enzyme activity comparison. $\mathbf{b_0}$, the induced *E. coli* BL21 (DE3) cell lysate; **b**, the cell lysate from induced BL21 (DE3) with original pET32a+. (**C**) Estimation of whole-cell transformation in comparison to crude enzyme activity. **ic**, *S.cerevisiae* intact cells; **r/ic**, the recombinant OYE2/pET32a+/BL21 (DE3) intact cells; **ce**, the crude extract of the recombinant OYE2/pET32a+/BL21 (DE3) cells.

induced BL21 (DE3) cell lysate from that of the induced original pET32a+/BL21 (DE3) cell lysate, we can estimate a 'net' fusion tag contribution to the reduction activity. By the same way we'll know if the entrokinase is active to geraniol. As shown in Figure 3B (columns c–e), neither fusion tag nor entrokinase was capable of forming citronellol from geraniol (Table S2 in Supplementary Material).

## Conclusions

In summary, we have first identified the enzyme responsible for the reduction of geraniol to citronellol. The recombinant HbOPR from rubber tree and the recombinant OYE2 from baker's yeast were found to be best performer and can be further prospected for their industrial biocatalysis applications. Despite of high protein level achieved by fusion expression of pET32a+, other expression vectors and *E. coli* strains may also be tried to harvest more enzymes. With the strict regioselectivity, the identified geraniol reductase should be further investigated for its stereospecificity and substrate specificity. Finally, we suggest the recombinant whole cells as biocatalysts in an economical way, considering the universal existence of NAD(P)H in living cells, the predominance of the enzymatic product citronellol, and almost no undesired side products.

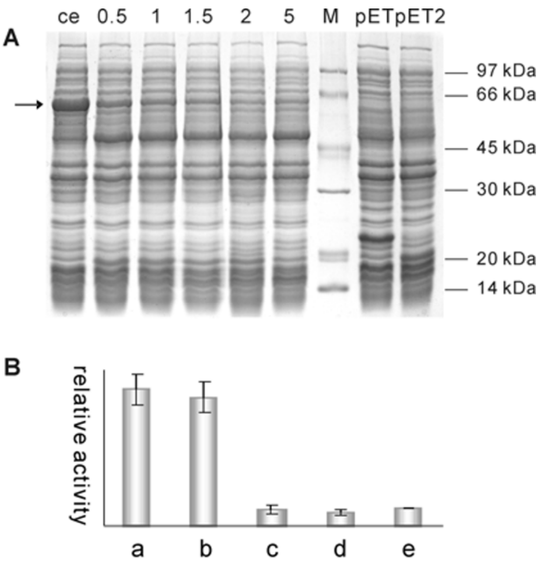

**Figure 3.** (**A**) Time course of fusion tag cleavage analyzed by SDS-PAGE. Numbers on the top line refer to incubation time (**h**) during the entrokinase cutting on the recombinant crude proteins. **ce**, crude enzyme extract containing the fusion protein (**arrow**) before entrokinase treatment; **M**, protein size marker; **pET**, the cell lysate containing the fusion tag from the induced pET32a+/BL21 (DE3); **pET2**, the cell lysate of induced pET32a+/BL21 (DE3) treated with entrokinase for 2 h. (**B**) Enzyme activity levels of the fusion protein and the entrokinase-cleaved protein. **a**, the cell lysate of recombinant HbOPR/pET32a+/BL21 (DE3); **b**, the entrokinase-cleaved cell lysate of recombinant HbOPR/pET32a+/BL21 (DE3); **c**, the cell lysate of pET32a+/BL21 (DE3); **d**, the cell lysate of BL21 (DE3); **e**, the entrokinase-treated cell lysate of BL21 (DE3).

## Experimental Section

**Gene Cloning.** Young leaves of *Epimedium acuminatum* and *Isodon eriocalyx* were collected from Kunming Botanic Garden, Chinese Academy of Sciences. Young shoots of *Hevea brasiliensis* were collected from Hekou County of southeast Yunnan province. The full length cDNAs of *EaOPR* and *IeOPR* were obtained by a homology-based cloning strategy using Superscript$^{TM}$ III First-strand Synthesis System and GeneRacer$^{TM}$ Kit (Invitrogen), and deposited in the GenBank/EMBL database under accession numbers JN828575 (for *EaOPR*) and JN828576 (for *IeOPR*), respectively. The open reading frame (ORF) of *HbOPR* was cloned by RT-PCR according to the sequence [GenBank: DQ004685.1]; and the ORF of *OYE2* [GenBank: NM_001179310.1] was amplified from the genomic DNA of *Saccharomyces cerevisiae*. All primers were listed in Table S1 in Supplementary Material.

**Expression in *E. coli*.** Each ORF of the four OYE genes was cloned into the expression vector pET32a+ (Novagen) which were subsequently transformed into *E. coli* BL21 (DE3) for a fusion expression, using the original pET32a+ as negative control. Protein expression at 37°C or 20°C with 0.1, 0.2 and 0.8 mM IPTG was determined by SDS-PAGE analysis. For crude enzyme preparation, cells induced with 0.1 mM IPTG at 20°C for 23 h were collected by centrifugation (8,000 rpm, 4°C, 5 min), washed by deionized water and suspended in 50 mM Tris-HCl pH7.5, 0.1M NaCl. For enzyme purification, the *E. coli* cells expressing HbOPR were induced and harvested as described above but suspended in the binding buffer (20 mM Tris-HCl pH7.5, 0.3 M NaCl, 5 mM imidazol). The cell lysate obtained by sonication on ice was then centrifuged at 12,000 rpm for 10 min at 4°C and the supernatant, containing the soluble recombinant enzyme was used for crude enzyme assay or purification. His-tagged proteins were purified to near homogeneity according to the Ni-NTA His•Bind Superflow protocol (Novagen) with a wash buffer (20 mM Tris-HCl pH7.5, 0.5 M NaCl, 50 mM imidazol) and an elution buffer (20 mM Tris-HCl pH7.5, 0.5 M NaCl, 0.2 M imidazol). All fractions were analyzed by SDS-PAGE on 12% polyacrylamide gel under non-reduced condition.

**Enzyme Assays and GC-MS Analyses.** Crude extracts of the induced BL21 (DE3) cells were assayed for enzyme activity using 1 mM of the corresponding substrate geraniol, methyl crotonate, methyl *trans*-cinnamate, and 3-methyl-2-cyclohexenone (all purchased from Sigma), incubating for 8 h at 37°C. No additional enzyme cofactor NADH or NADPH was needed since the crude extracts contain both. After extraction with ethyl acetate, the upper phase was collected by centrifugation, dehydrated over anhydrous sodium sulfate and concentrated under a stream of $N_2$ for GC-MS analysis. The identification of the enzyme product was achieved by comparing the retention time and the mass spectra with those of the authentic compound.

In the following enzymatic assays performed in duplicate, each reaction containing 100 $\mu$L of crude extracts (or intact cells) from an equal amount of cells (calculated by the value of $OD_{600}$ × volume of the culture) was incubated with 1 mM geraniol at 37°C for 4 h. A precise amount (4.88 $\mu$g) of methyl *trans*-cinnamate was added as an internal standard in each reaction mixture after incubation. Relative enzyme activity was estimated from the ratio of GC integral of citronellol with respect to the internal standard. For enzymatic activity level comparison, reactions carried out with crude extracts were analyzed under the same condition, using the induced BL21 (DE3) cell lysate and the lysate from induced cells harboring the original pET32a+ as controls. For estimation of whole-cell transformation in comparison to crude enzyme activity, we used an equal amount of *S. cerevisiae* yeast cells, the induced OYE2/pET32a+/*E. coli* BL21 (DE3) intact cells, and the crude extracts from the same amount of induced OYE2/pET32a+/*E. coli* BL21 (DE3) cells. For fusion tag cleavage, the induced HbOPR/pET32a+/BL21 (DE3) cells sonicated in 100 $\mu$L buffer (50 mM Tris-HCl pH7.5, 0.1 M NaCl, 2 mM $CaCl_2$) was cleaved with 40 IU entrokinase (Shanghai, Sangon) at RT (24~27°C) for 0.5, 1, 1.5, 2, 5 h, and subsequently analyzed by SDS-PAGE. The completely cleaved (2 h) protein was assayed for enzyme activity as descried above. Meanwhile, crude extracts (kept for 2h before enzyme assay) from the induced HbOPR cells and the induced cells harboring the original pET32a+, together with the induced BL21 (DE3) cell lysate prepared with or without entrokinase were analyzed in parallel to serve as controls. For cofactor determination, the purified recombinant HbOPR enzyme was incubated at 37°C for 4h in the reaction mixture containing 1mM geraniol and 3 mM NADH or NADPH.

GC-MS [Agilent HP6890/5973, column: 0.25 mm × 30 m, 0.25 $\mu$m (HP-5MS)] was conducted under electron-impact (EI) mode (70 eV). The flow rate of helium carrier gas was set at 1.0 ml/min. Samples (2 $\mu$L) were injected at 60°C. After holding the samples for 5 min at 60°C, the column temperature was increased at 10°C/min to 260°C and hold for 10 min. The MS date was collected from 35 to 450 *m/z*.

## Electronic Supplementary Material

Supplementary material is available in the online version of this article at http://dx.doi.org/10.1007/s13659-011-0032-6 and is accessible for authorized users.

## Acknowledgments

This work was supported by grants from the Ministry of Science and Technology of China (2007CB108802) and State Key Laboratory of Phytochemistry and Plant Resources in West China (P2008-ZZ16, P2009-ZZ02).

**Open Access** This article is distributed under the terms of the Creative Commons Attribution License which permits any use, distribution, and reproduction in any medium, provided the original author(s) and source are credited.

## Electronic supplementary material

Supplementary material, approximately 398 KB.
