# Supplementary material for: Identification of enzymes responsible for the reduction of geraniol to citronellol
Source: Nat Prod Bioprospect. 2011 Dec 13;1(3):108–11. doi: 10.1007/s13659-011-0032-6 (PMC4131645; doi:10.1007/s13659-011-0032-6)
Supplement: Supplementary file 1 — Supplementary material, approximately 398 KB. [file 13659_2011_32_MOESM1_ESM.pdf]

Electronic Supplementary Material

Identification of enzymes responsible for the reduction of geraniol to citronellol

Tian-Tian YUAN,[a,b] Qian-Qian CHEN,[a] Pei-Ji ZHAO,[a] Ying ZENG,[a,]* Xiao-Zhu LIU,[b] and Shan LU[c]

[a]State Key Laboratory of Phytochemistry and Plant Resources in West China, Kunming Institute of Botany, Chinese Academy of Sciences, Kunming 650201, China

[b]Life Science College, Southwest Forestry University, Kunming 650224, China

[c]State Key Laboratory of Pharmaceutical Biotechnology, Nanjing University, Nanjing 210093, China

Received 17 November 2011; Accepted 30 November 2011

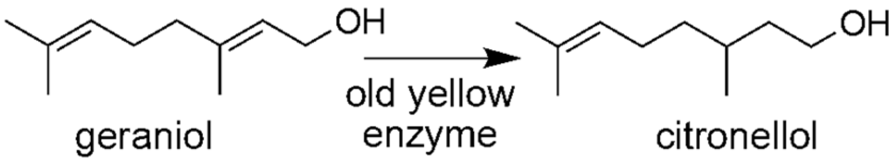

Scheme. The enzyme-catalyzed reduction of geraniol to citronellol.

*To whom correspondence should be addressed. E-mail: biochem@mail.kib.ac.cn.

Content list:

Figure S1. SDS-PAGE analyses of crude extracts from the recombinant HbOPR (A) and yeast OYE2 (B) heterologously expressed in E. coli BL21 (DE3) at 37°C/ 0.1/ 0.2/ 0.8 mM IPTG (from left to right) and 20°C/ 0.1/ 0.2 mM IPTG (from left to right), respectively. 1, whole cell lysate before centrifugation; 2, the cleared cell lysate; M, protein size marker; pET, extracts of E. coli with original pET32a+ as control.

Figure S2. GC-MS analyses of the product 3-methyl-2-cyclohexanone (RT=4.02 min) formed by the crude recombinant OYE2 enzyme with 3-methyl-2-cyclohexenone (RT=5.71 min) as substrate (11082702.D), using the cell lysate of BL21 (DE3) containing original pET32a+ as the control (11082700.D).

Figure S3. GC-MS analyses of the crude enzyme assay products formed by the recombinant EaOPR (11092400.D), HbOPR (11092403.D), IeOPR (11092401.D) and OYE2 (11092601.D), respectively, using the cell lysate of BL21 (DE3) containing original pET32a+ as control (11090301.D) and the authentic citronellol, geraniol and methyl trans-cinnamate (the internal standard) as reference (11090102.D, 11100706.D).The retention time of the product citronellol, the substrate geraniol and the internal standard peaks at 9.29   9.36 min, 9.95   10.06 min and 13.21~13.26 min, respectively.

Figure S4. GC-MS analyses of whole-cell transformation vs crude enzyme activity including S.cerevisiae yeast cells (11092806.D), the induced OYE2/pET32a+/BL21 (DE3) intact cells (11092802.D), and the crude recombinant OYE2 extracts (11092804.D).

Figure S5. GC-MS analyses of the crude enzyme products formed by the recombinant HbOPR as a fusion protein (11090201.D) and a 'net' protein (11090202.D), respectively. The retention time of the product citronellol peaks at 9.33 min, and the internal standard at 13.25 min.

Table S1. Degenerate primers for cloning EaOPR and IeOPR and primer pairs for cloning EaOPR, HbOPR, IeOPR and OYE2 into pET32a+.

Table S2. Primary data for enzyme activity comparison, estimation of whole-cell transformation vs crude enzyme activity, and fusion tag cleavage.

Figure S1. SDS-PAGE analyses of crude extracts from the recombinant HbOPR (**A**) and yeast OYE2 (**B**) heterologously expressed in *E. coli* BL21 (DE3) at 37°C/ 0.1/ 0.2/ 0.8 mM IPTG (from left to right) and 20°C/ 0.1/ 0.2 mM IPTG (from left to right), respectively. **1,** whole cell lysate before centrifugation; **2,** the cleared cell lysate; **M,** protein size marker; **pET,** extracts of *E. coli* with original pET32a+ as control.

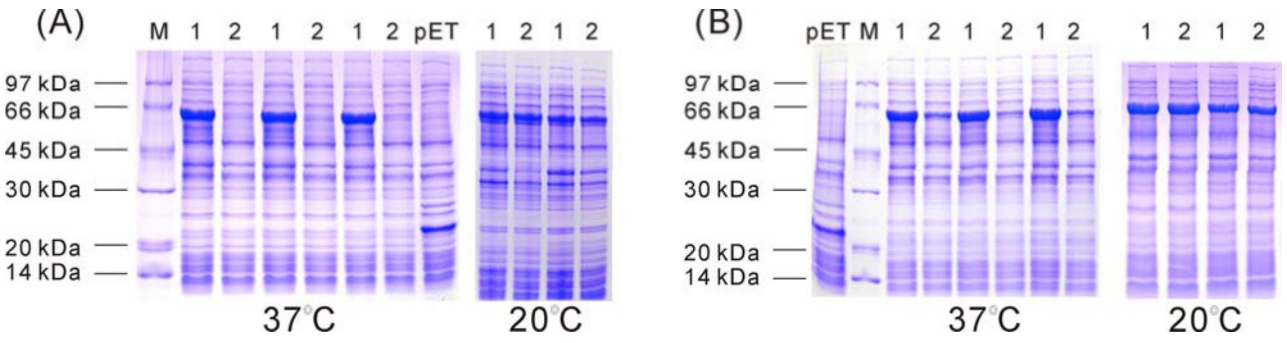

Figure S2. GC-MS analyses of the product 3-methyl-2-cyclohexanone (RT=4.02 min) formed by the crude recombinant OYE2 enzyme with 3-methyl-2-cyclohexenone (RT=5.71 min) as substrate (11082702.D), using the cell lysate of BL21 (DE3) containing original pET32a+ as the control (11082700.D).

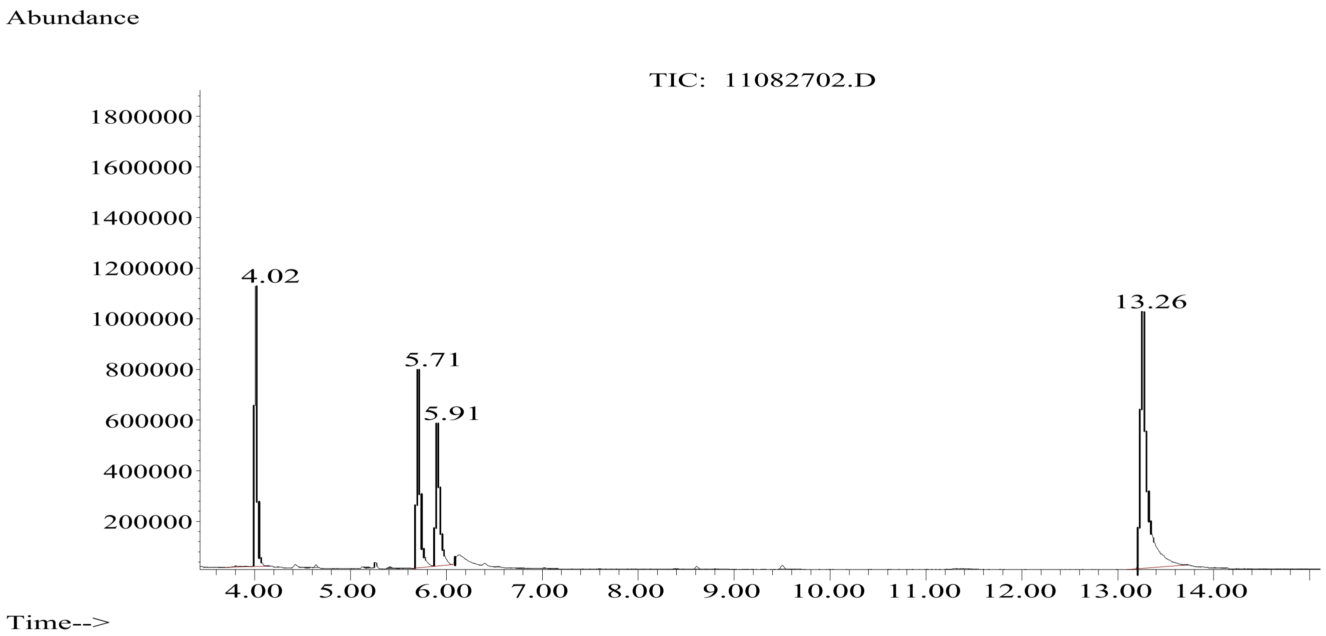

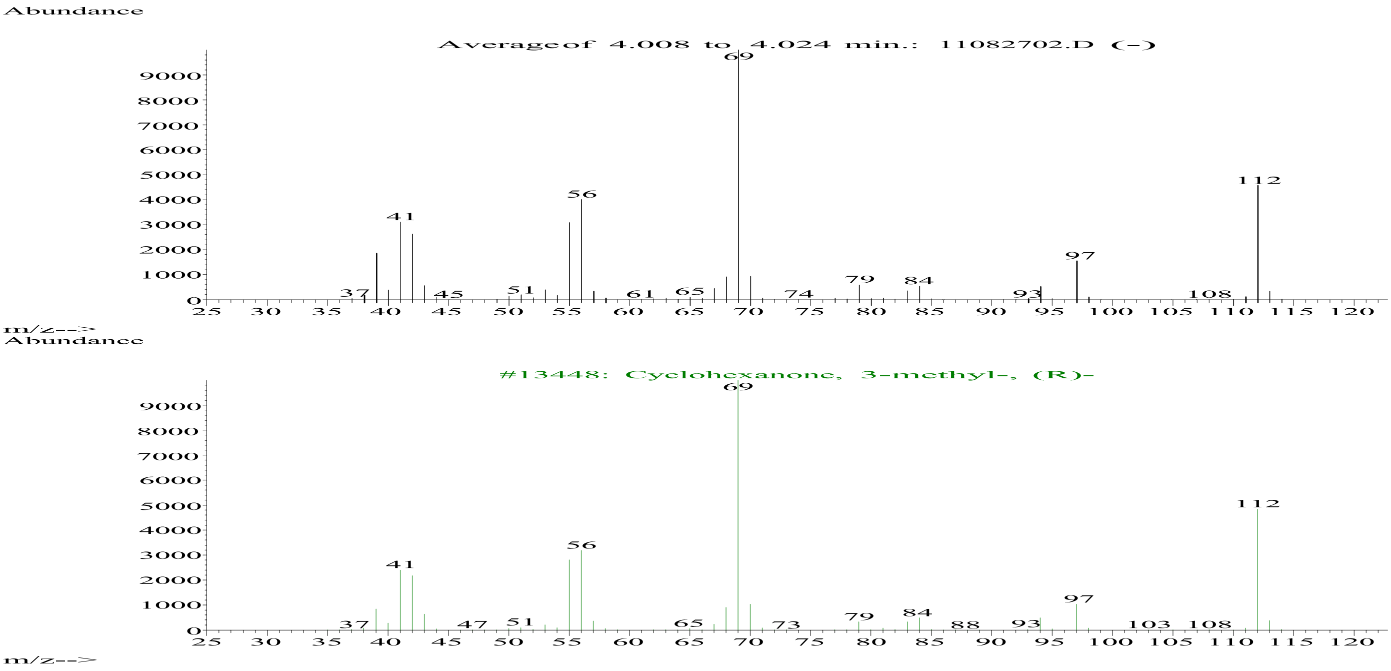

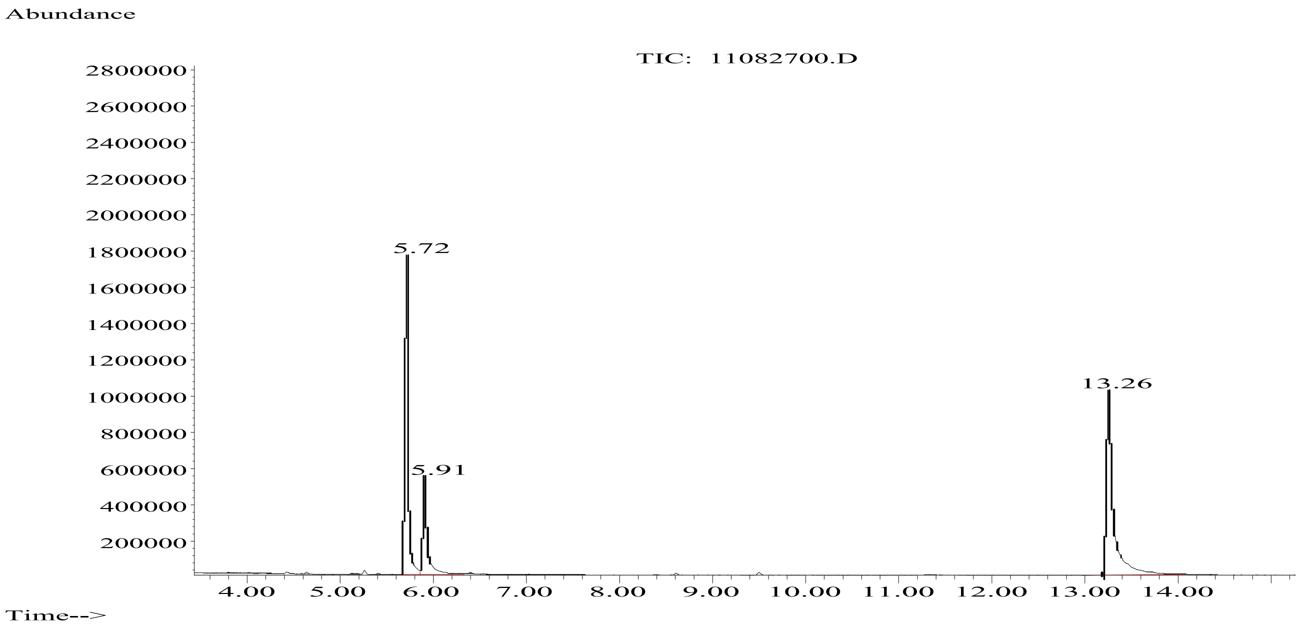

Figure S3. GC-MS analyses of the crude enzyme assay products formed by the recombinant EaOPR (11092400.D), HbOPR (11092403.D), IeOPR (11092401.D) and OYE2 (11092601.D), respectively, using the cell lysate of BL21 (DE3) containing original pET32a+ as control (11090301.D) and the authentic citronellol, geraniol and methyl *trans*-cinnamate (the internal standard) as reference (11090102.D, 11100706.D).The retention time of the product citronellol, the substrate geraniol and the internal standard peaks at 9.29~9.36 min, 9.95~10.06 min and 13.21~13.26 min, respectively.

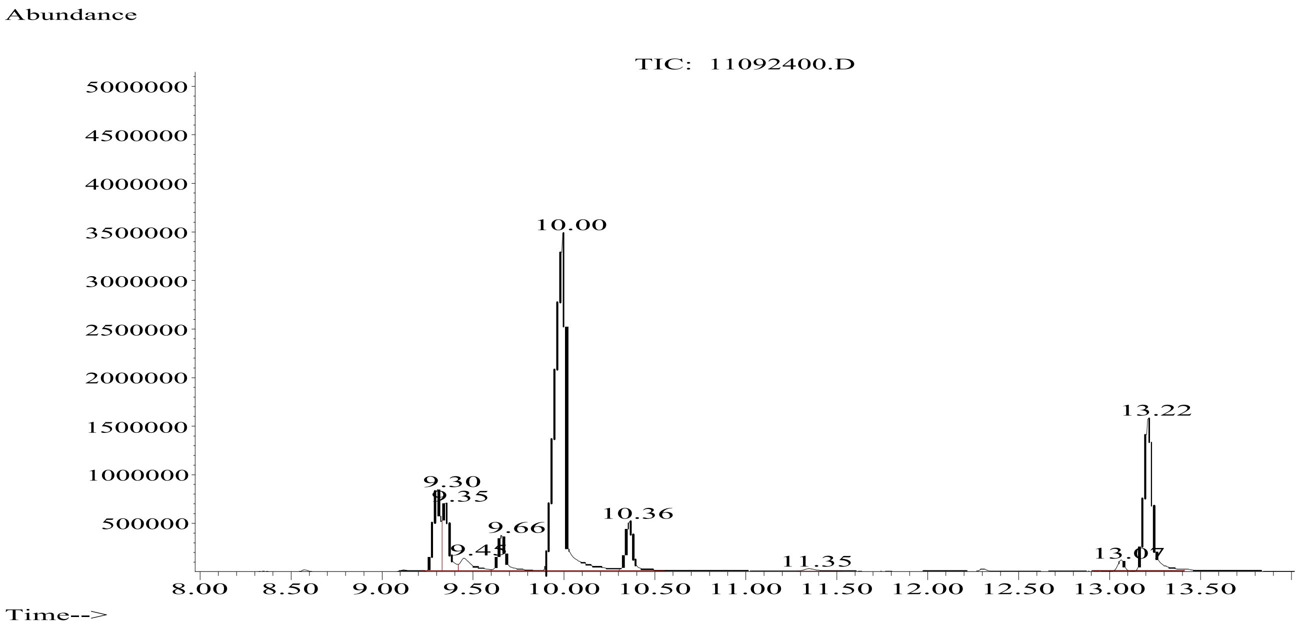
Abundance
TIC: 11092400.D
5000000
4500000
4000000
3500000
3000000
2500000
2000000
1500000
1000000
500000
10.00
13.22
9.30
9.35
9.43
9.66
10.36
11.35
13.07
8.00 8.50 9.00 9.50 10.00 10.50 11.00 11.50 12.00 12.50 13.00 13.50
Time-->

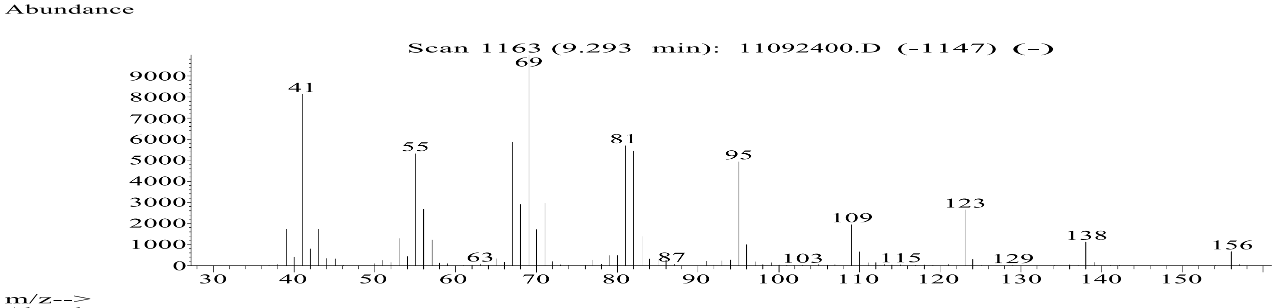
Abundance
Scan 1163 (9.293 min): 11092400.D (-1147) (-)
41
55
63
69
81
87
95
103
109
115
123
129
138
156
30 40 50 60 70 80 90 100 110 120 130 140 150
m/z-->

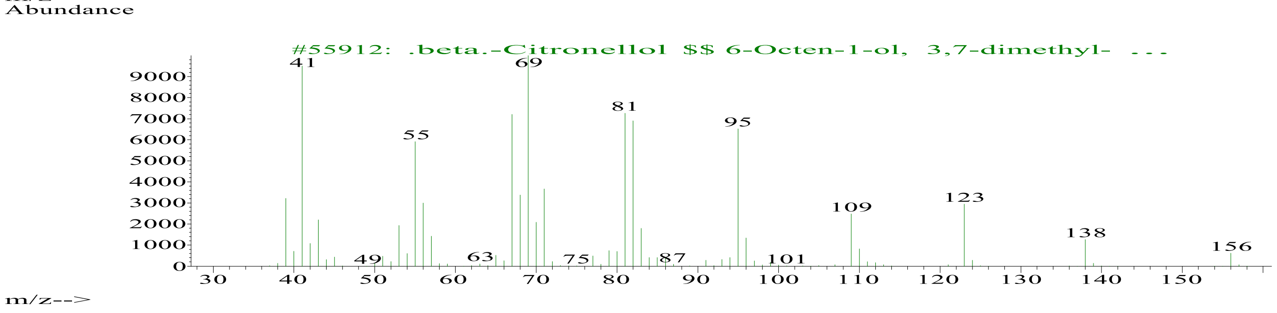
Abundance
#55912: .beta.-Citronellol $$ 6-Octen-1-ol, 3,7-dimethyl- ...
41
49
55
63
69
75
81
87
95
101
109
123
138
156
30 40 50 60 70 80 90 100 110 120 130 140 150
m/z-->

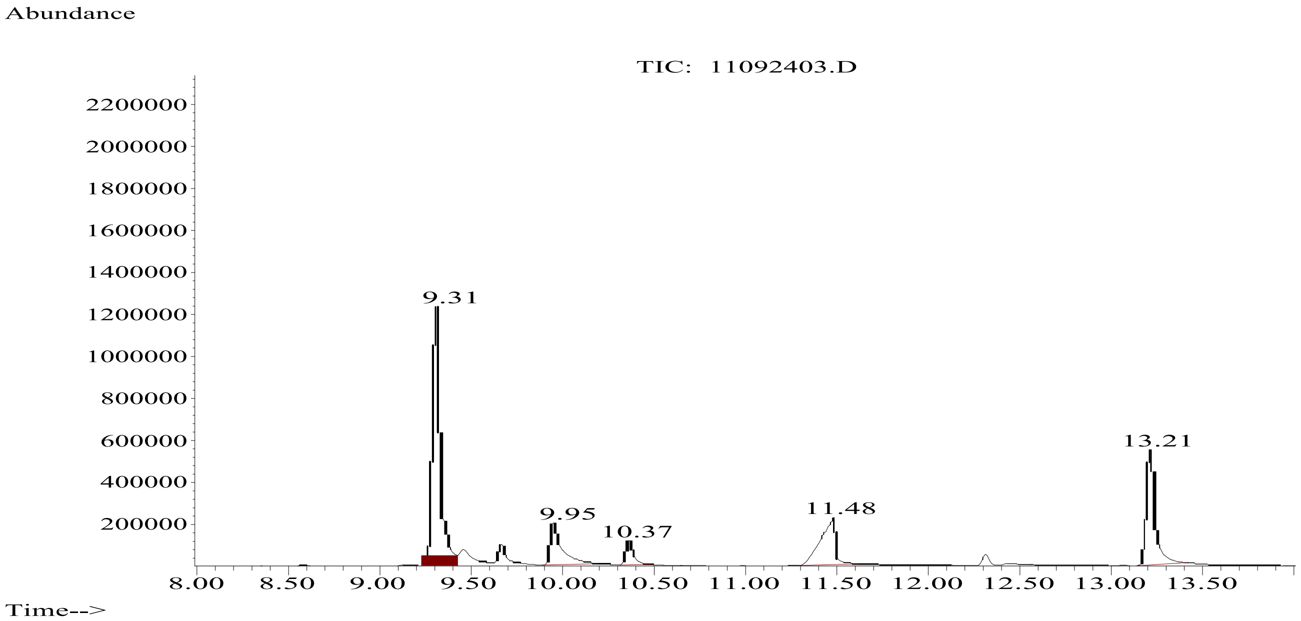
Abundance
TIC: 11092403.D
2200000
2000000
1800000
1600000
1400000
1200000
1000000
800000
600000
400000
200000
9.31
9.95
10.37
11.48
13.21
8.00 8.50 9.00 9.50 10.00 10.50 11.00 11.50 12.00 12.50 13.00 13.50
Time-->

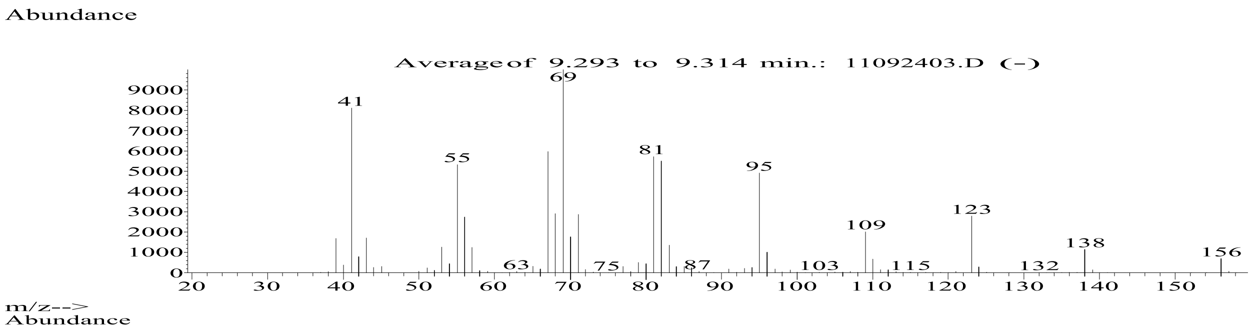
Abundance
Average of 9.293 to 9.314 min.: 11092403.D (-)
41
55
69
81
95
109
123
138
156
m/z-->

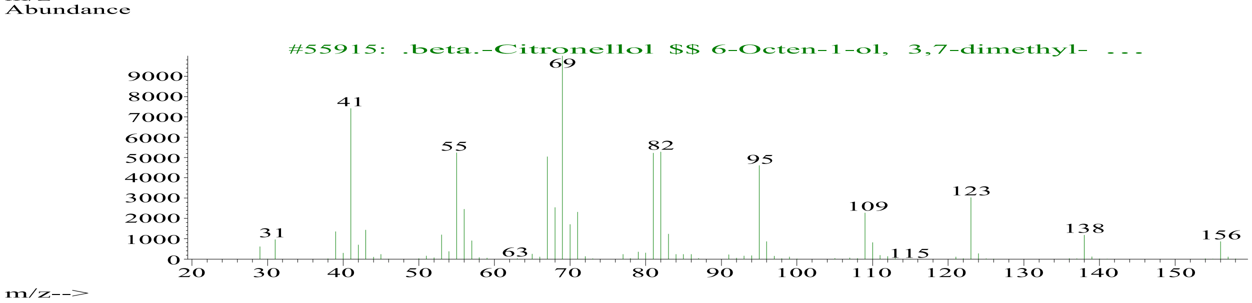
Abundance
#55915: .beta.-Citronellol $$ 6-Octen-1-ol, 3,7-dimethyl- ...
41
55
69
82
95
109
123
138
156
m/z-->

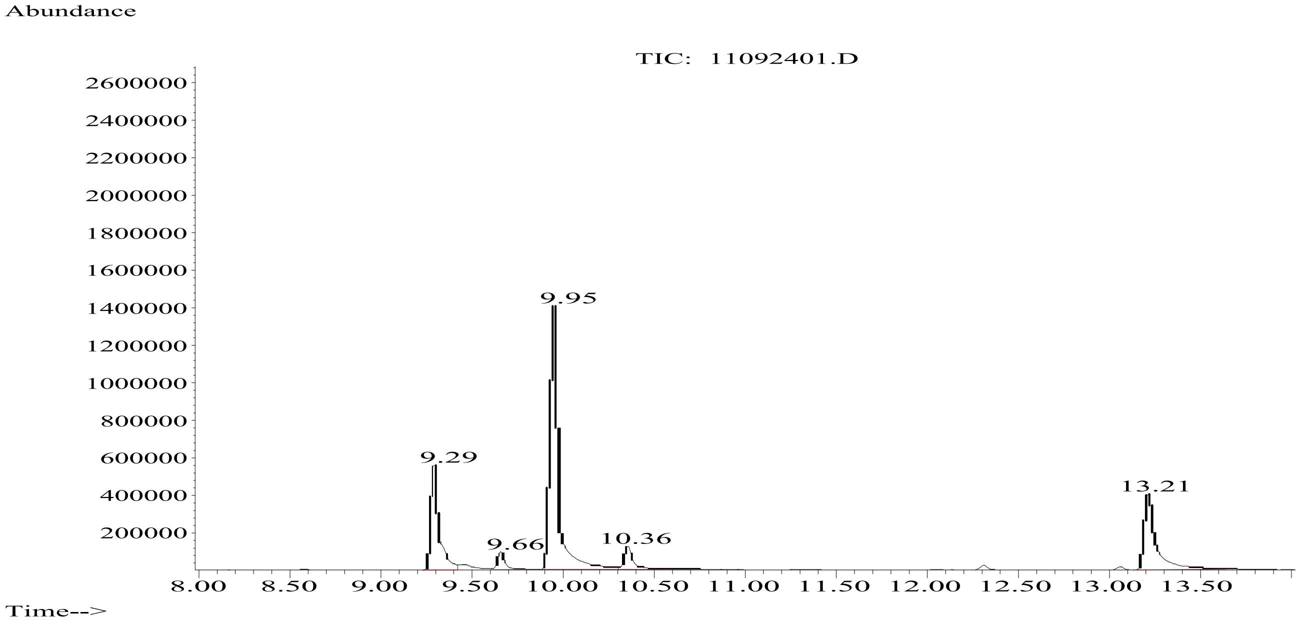
Abundance
TIC: 11092401.D
9.29
9.66
9.95
10.36
13.21
Time-->

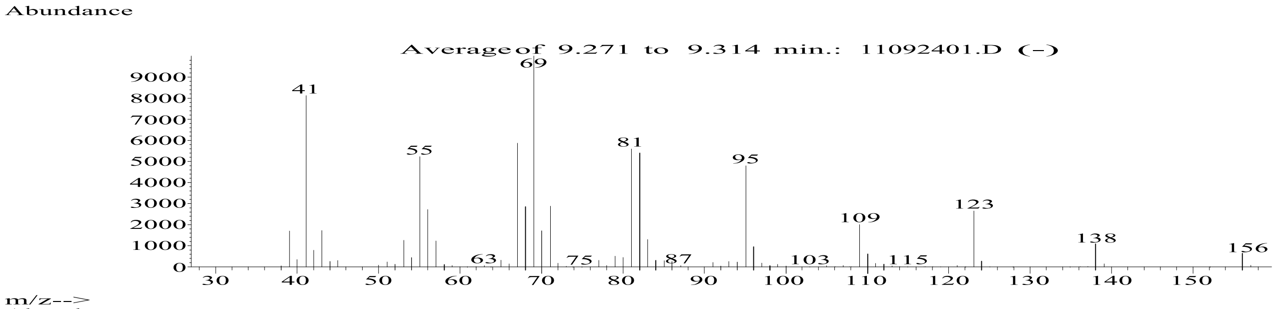
Abundance
Average of 9.271 to 9.314 min.: 11092401.D (-)
41
55
69
81
95
109
123
138
156
m/z-->

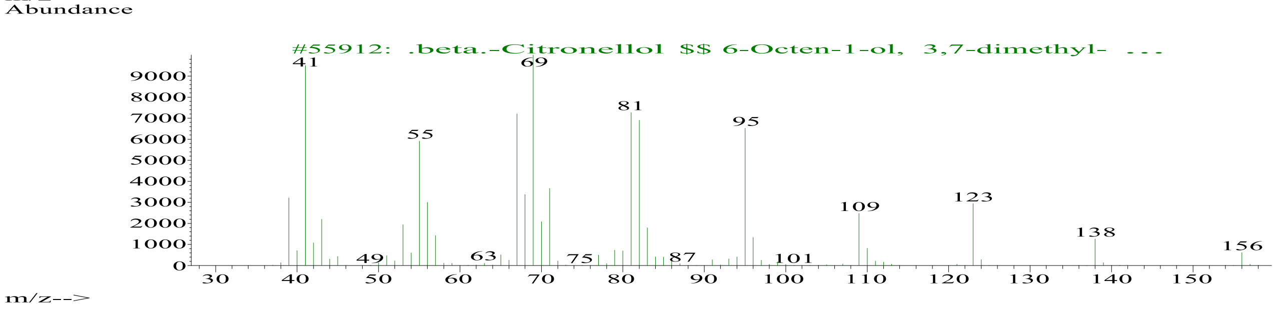
Abundance
#55912: .beta.-Citronellol $$ 6-Octen-1-ol, 3,7-dimethyl- ...
41
55
69
81
95
109
123
138
156
m/z-->

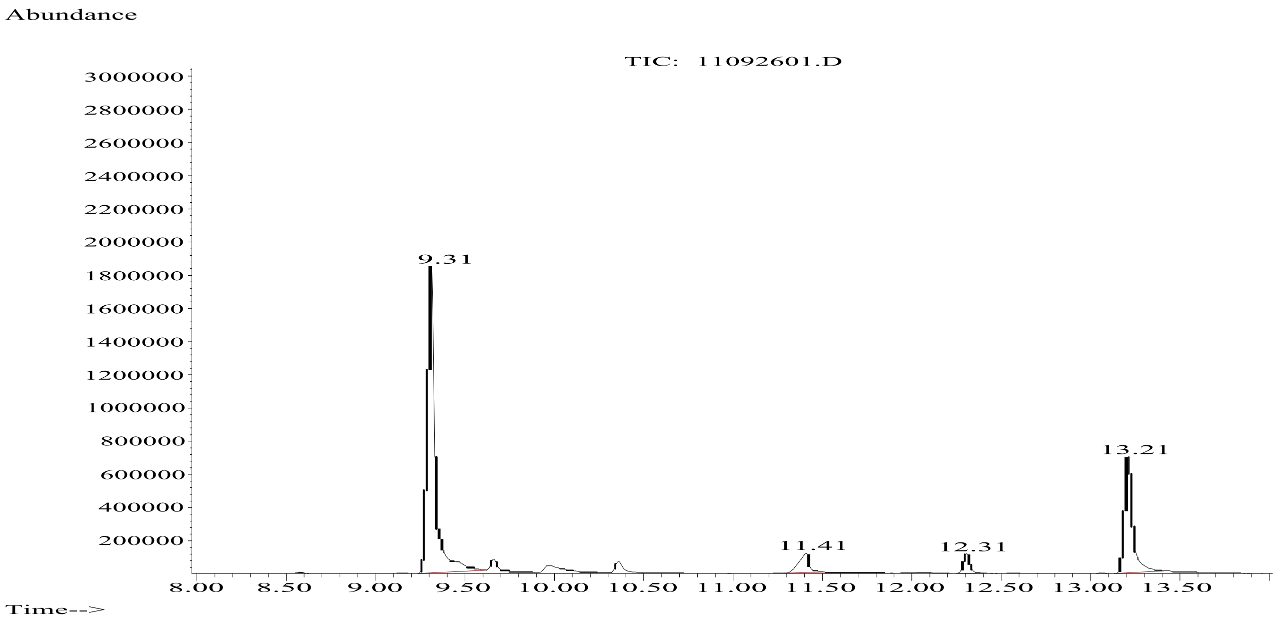
Abundance
TIC: 11092601.D
3000000
2800000
2600000
2400000
2200000
2000000
1800000
1600000
1400000
1200000
1000000
800000
600000
400000
200000
9.31
11.41
12.31
13.21
8.00 8.50 9.00 9.50 10.00 10.50 11.00 11.50 12.00 12.50 13.00 13.50
Time-->

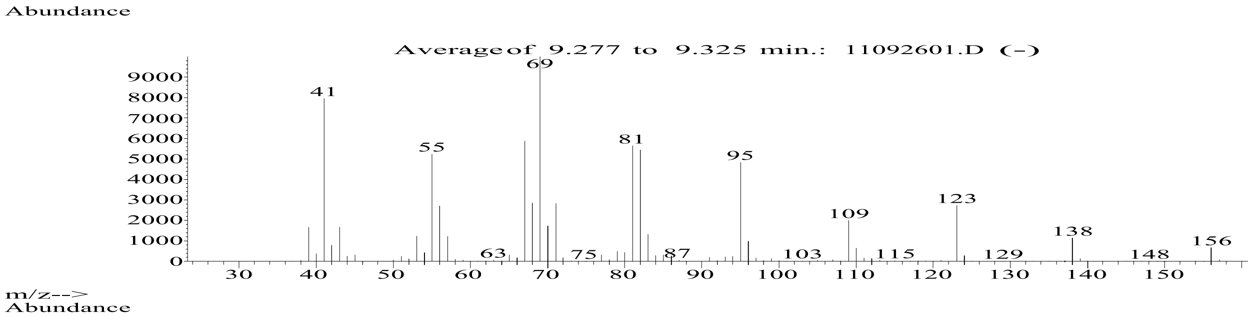
Abundance
Average of 9.277 to 9.325 min.: 11092601.D (-)
9000
8000
7000
6000
5000
4000
3000
2000
1000
0
41
55
63
69
75
81
87
95
103
109
115
123
129
138
148
156
30 40 50 60 70 80 90 100 110 120 130 140 150
m/z-->

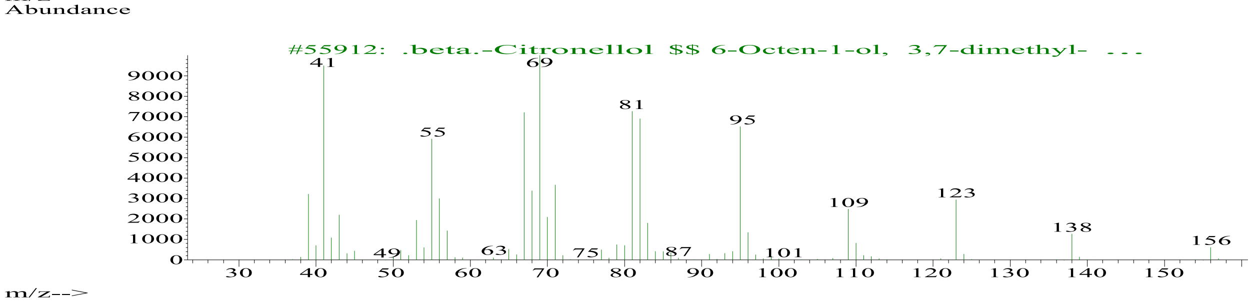
Abundance
#55912: .beta.-Citronellol $$ 6-Octen-1-ol, 3,7-dimethyl- ...
9000
8000
7000
6000
5000
4000
3000
2000
1000
0
41
49
55
63
69
75
81
87
95
101
109
123
138
156
30 40 50 60 70 80 90 100 110 120 130 140 150
m/z-->

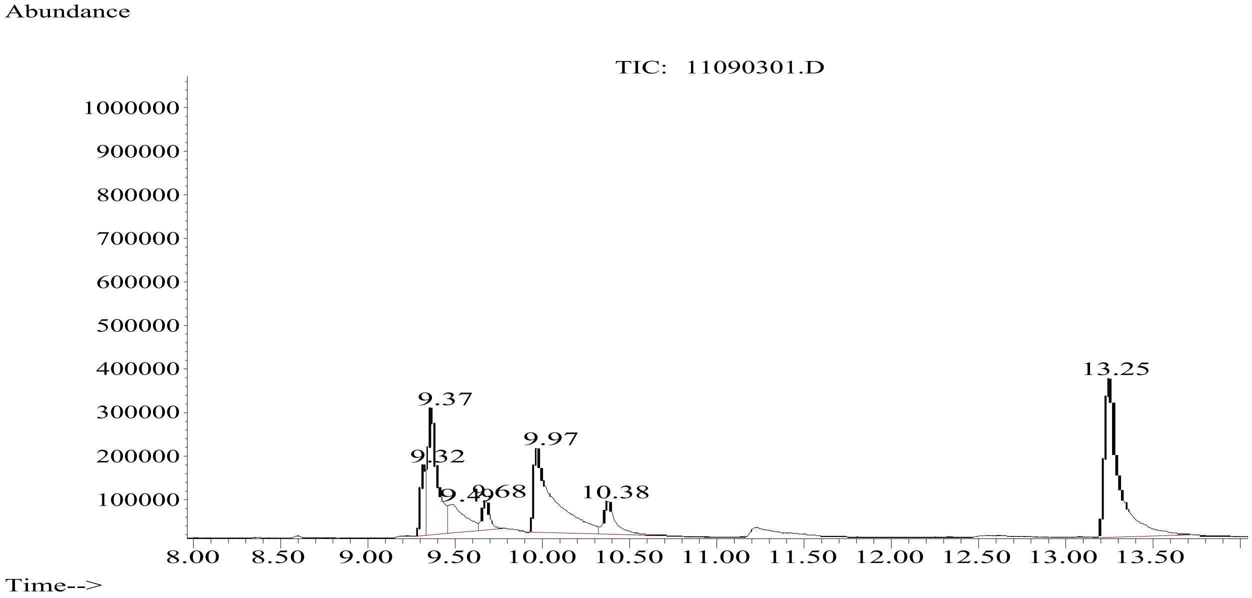
Abundance
TIC: 11090301.D
1000000
900000
800000
700000
600000
500000
400000
300000
200000
100000
9.37
9.32
9.49
9.68
9.97
10.38
13.25
8.00 8.50 9.00 9.50 10.00 10.50 11.00 11.50 12.00 12.50 13.00 13.50
Time-->

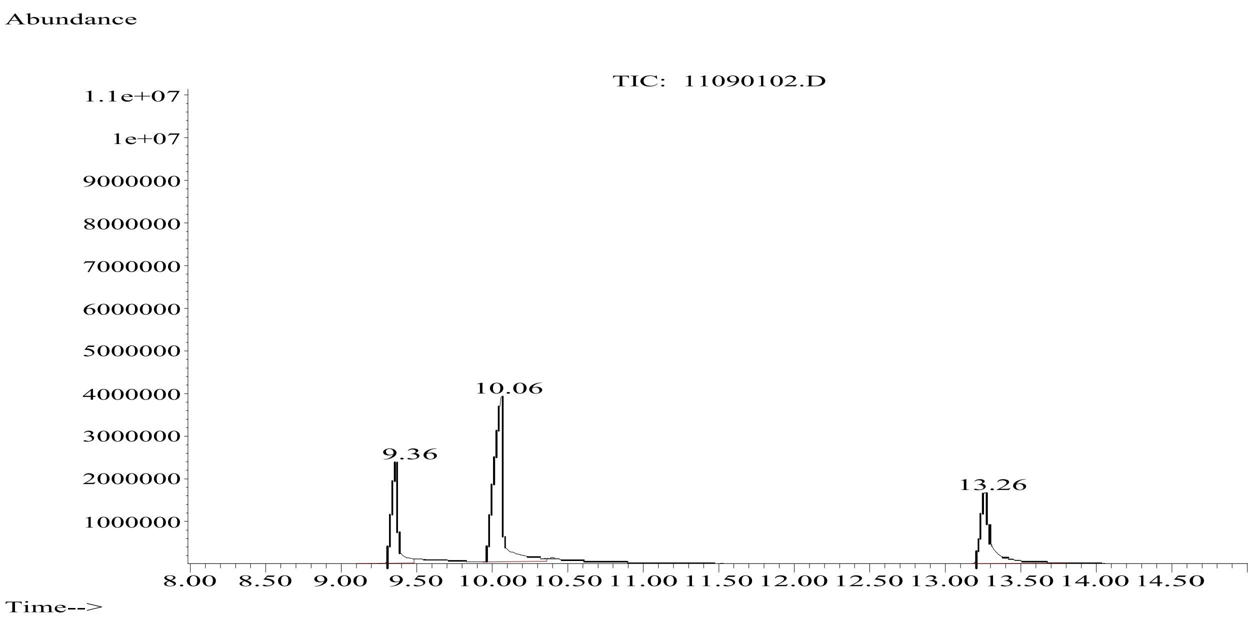
Abundance
TIC: 11090102.D
1.1e+07
1e+07
9000000
8000000
7000000
6000000
5000000
4000000
3000000
2000000
1000000
9.36
10.06
13.26
8.00 8.50 9.00 9.50 10.00 10.50 11.00 11.50 12.00 12.50 13.00 13.50 14.00 14.50
Time-->

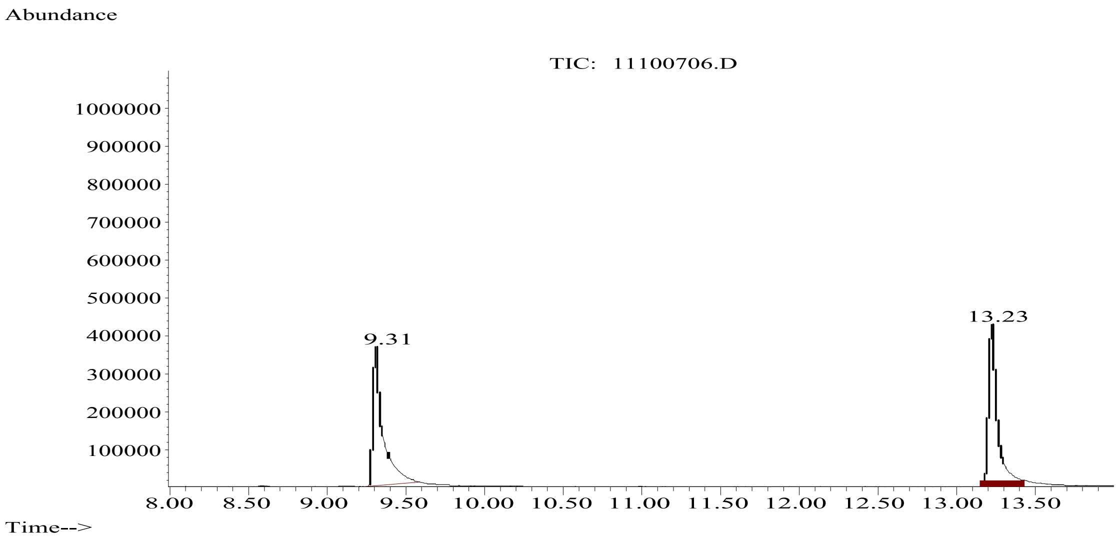
Abundance
TIC: 11100706.D
1000000
900000
800000
700000
600000
500000
400000
300000
200000
100000
9.31
13.23
8.00 8.50 9.00 9.50 10.00 10.50 11.00 11.50 12.00 12.50 13.00 13.50
Time-->

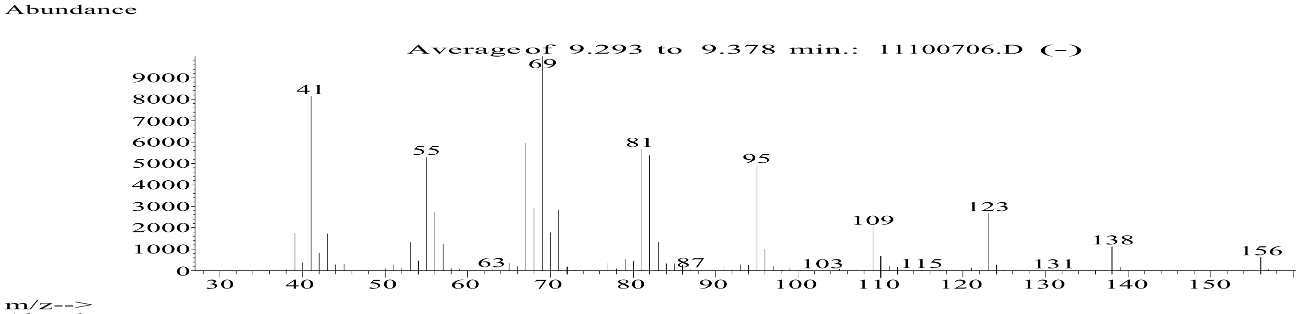
Abundance
Average of 9.293 to 9.378 min.: 11100706.D (-)
9000
8000
7000
6000
5000
4000
3000
2000
1000
0
41
55
63
69
81
87
95
103
109
115
123
131
138
156
30 40 50 60 70 80 90 100 110 120 130 140 150
m/z-->

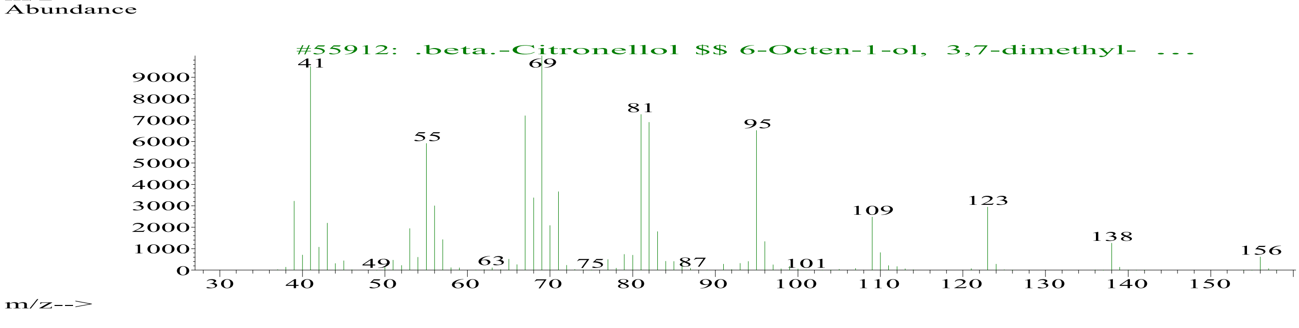
Abundance
#55912: .beta.-Citronellol $$ 6-Octen-1-ol, 3,7-dimethyl- ...
9000
8000
7000
6000
5000
4000
3000
2000
1000
0
41
49
55
63
69
75
81
87
95
101
109
123
138
156
30 40 50 60 70 80 90 100 110 120 130 140 150
m/z-->

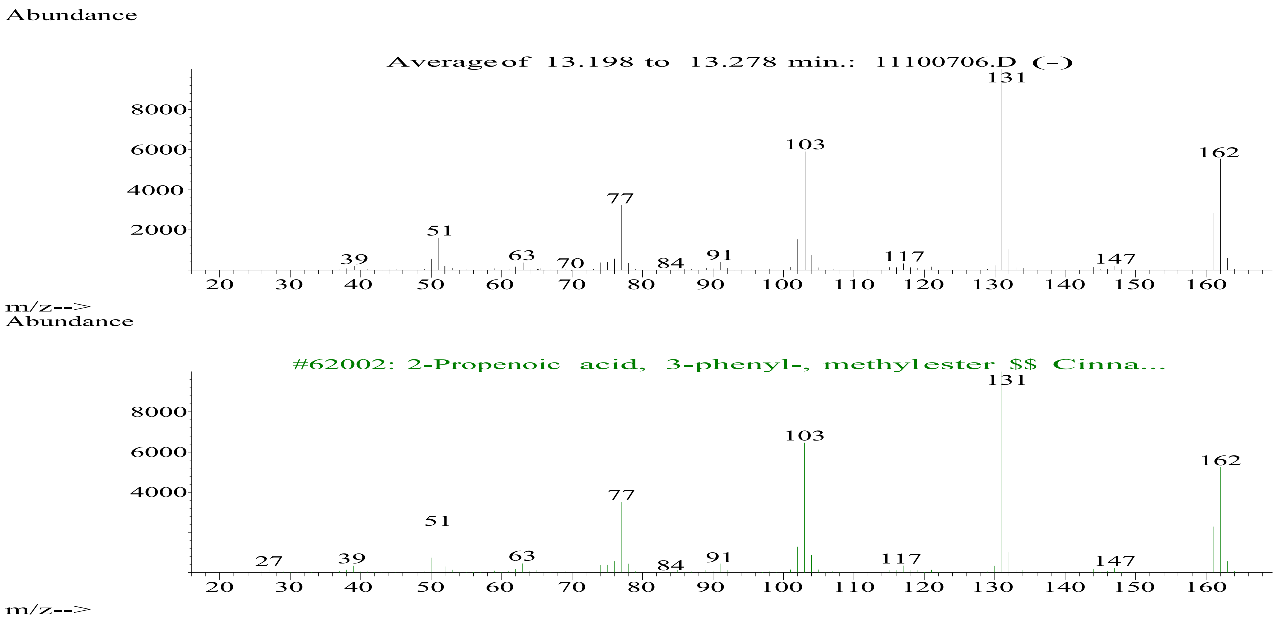

Figure S4. GC-MS analyses of whole-cell transformation *vs* crude enzyme activity including *S.cerevisiae* yeast cells (11092806.D), the induced OYE2/pET32a+/BL21 (DE3) intact cells (11092802.D), and the crude recombinant OYE2 extracts (11092804.D).

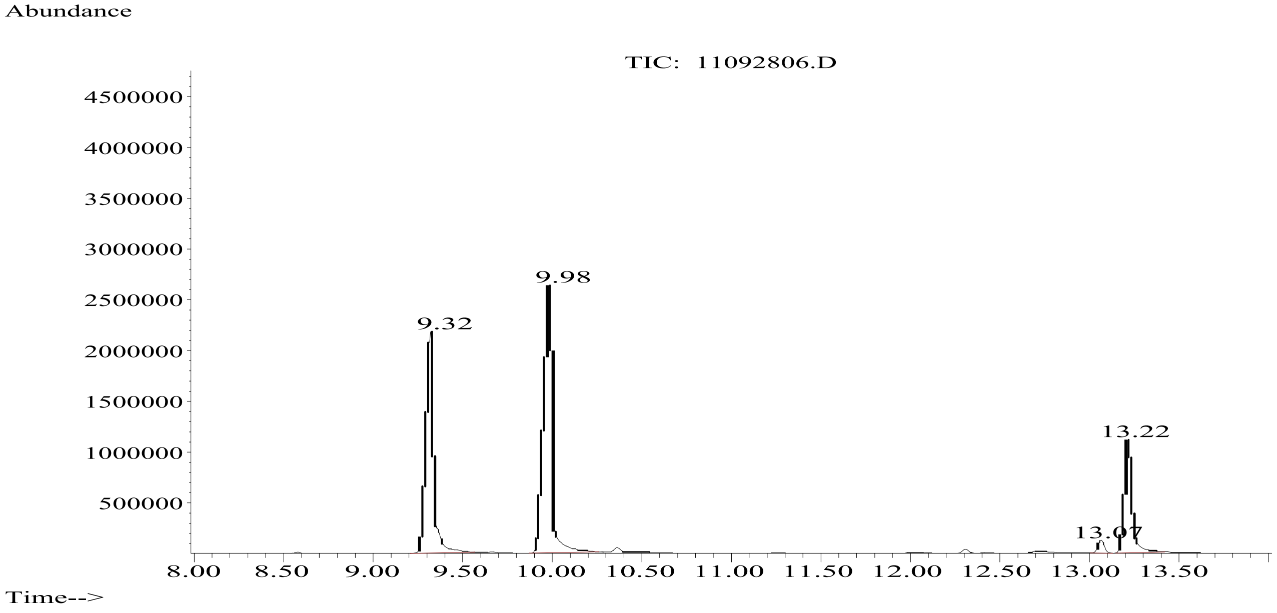

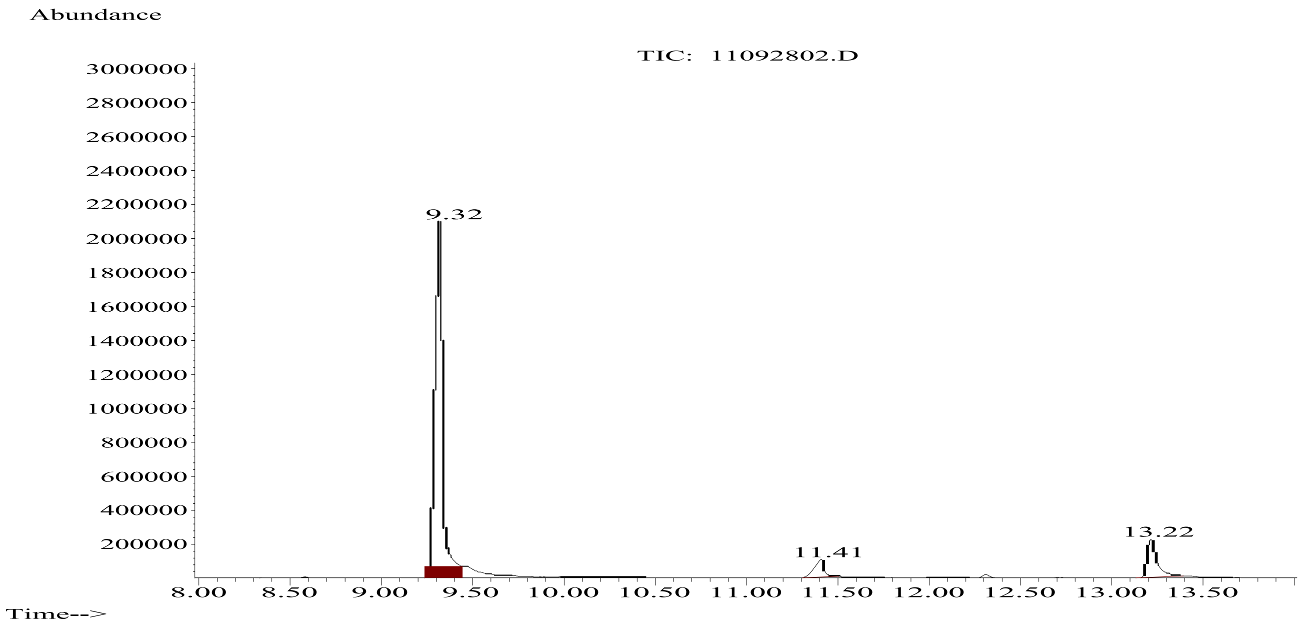

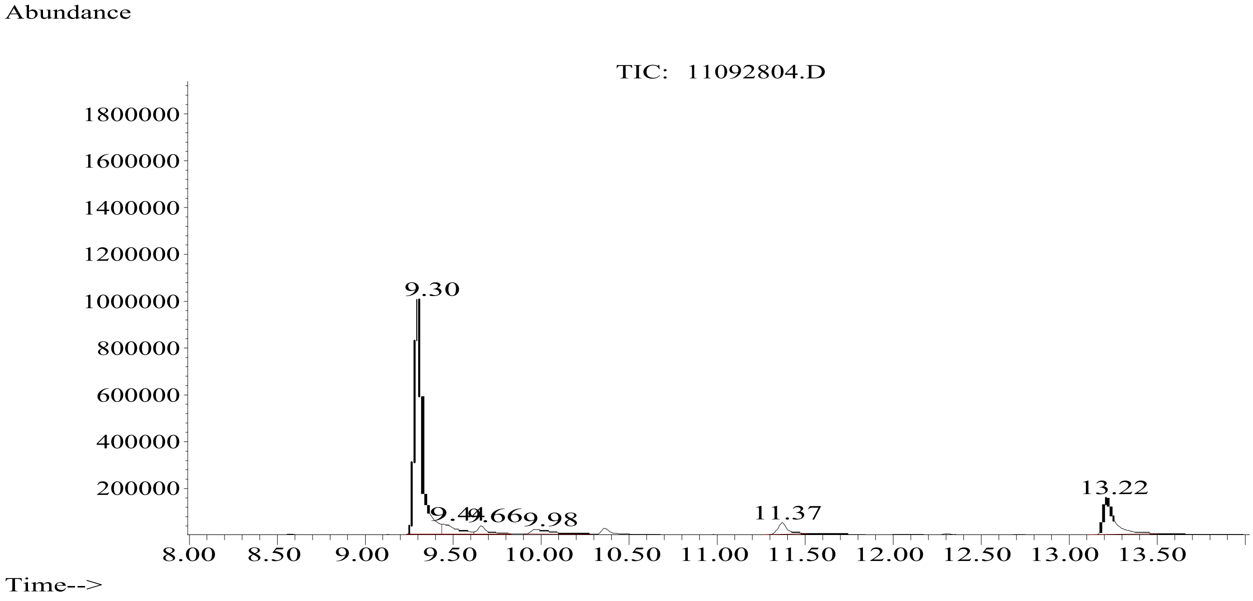

Figure S5. GC-MS analyses of the crude enzyme products formed by the recombinant HbOPR as a fusion protein (11090201.D) and a 'net' protein (11090202.D), respectively. The retention time of the product citronellol peaks at 9.33 min, and the internal standard at 13.25 min.

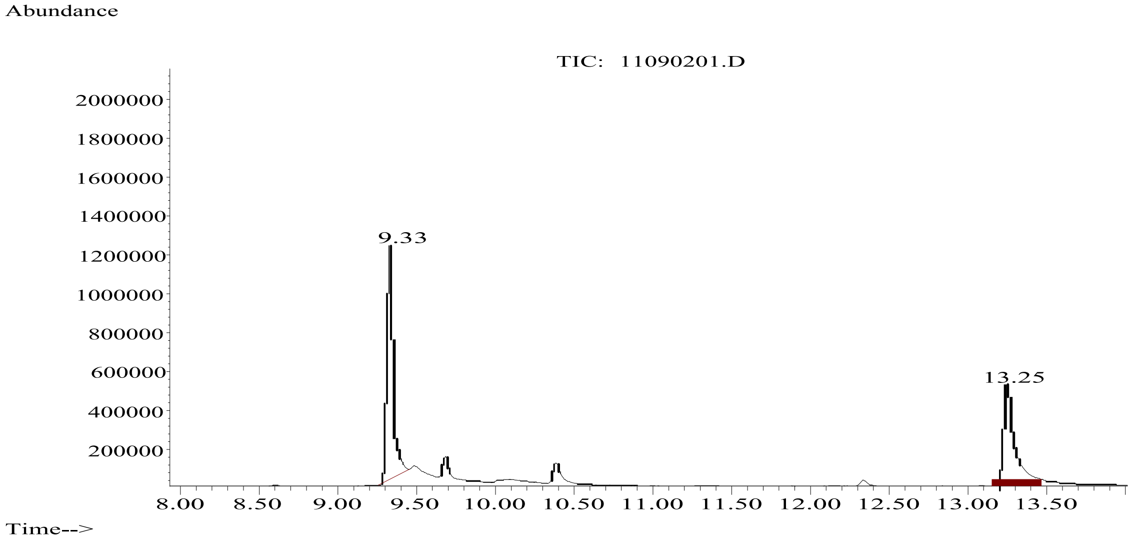

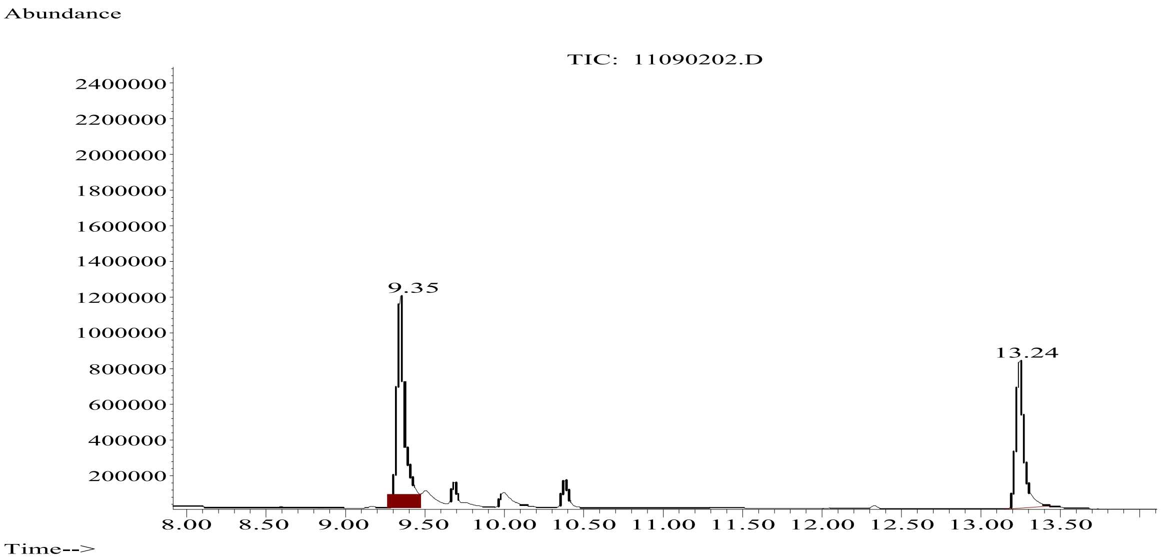

Table S1. Degenerate primers for cloning *EaOPR* and *IeOPR* and primer pairs for cloning *EaOPR, HbOPR, IeOPR* and *OYE2* into pET32a+

	Sequence (5´-3´)
Degenerate	Forward: CCYTACAARATGGGCAASTTC
Primers	Reverse: CCACCATAYTCRTCYGTYCKGTC
Ea	Forward: GGAATTCATGGGATCCTATACTGAATCG
	Reverse: CGCTCGAGTCAAGTCGAAATCTCAGAAG
Ie	Forward: TGAATTCATGGCGGAAACGACTCCCG
	Reverse: GACCTCGAGTTTAAAATCGCGATGCAGC
Hb	Forward: CGGATCCATGGCTGAAACTGGAACAG
	Reverse: GACCTCGAGTTCAAAGGCGTGATCGTGGCT
Sc	Forward: GGGAATTCATGCCATTTGTTAAGG
	Reverse: GGCCTCGAGTTTAATTTTTGTCCC

Table S2. Primary data for enzyme activity comparison, estimation of whole-cell transformation *vs* crude enzyme activity, and fusion tag cleavage

Comparison of Enzyme Activity Level

	**pET32a+ / BL21 (DE3)**	**BL21 (DE3)**	**EaOPR**	**HbOPR**	**IeOPR**	**ScOYE2**
**C / IS**	0.2256	0.2266	0.3777	2.3185	0.9142	2.3677
(GC integral)	0.2697	0.2320	0.4455	2.5584	1.0281	2.4010

Whole-Cell Transformation *vs* Crude Enzyme Activity

	Saccharomyces cerevisiae intact cells		OYE2/pET32a+/ BL21 (DE3) intact cells		OYE2/ pET32a+/ BL21 (DE3) cell lysates	
	28°C, 12h	37°C, 4h	28°C, 12h	37°C, 4h	28°C, 12h	37°C, 4h
**C / IS**	1.9131	1.1372	6.9931	4.4072	4.600	2.3677
(GC integral)	2.1113	1.2916	7.6456	4.5827	4.7840	2.4010

Fusion Tag Cleavage

	**fusion protein**	**EK-cleaved protein**	**pET32a+ / BL21 (DE3)**	**BL21 (DE3)**	**EK + BL21 (DE3)**	**EK + pET32a+/ BL21 (DE3)**
**C / IS**	1.21	1.35	0.13	0.11	0.17	0.15
(GC integral)	1.43	1.14	0.19	0.15	0.18	0.18

C, Citronellol; **IS**, internal standard (methyl *trans*-cinnamate)
